# Computed tomography as a solution to skeletal collection limitations: exploring cranial variation in the South African population

**DOI:** 10.1007/s00414-026-03878-y

**Published:** 2026-06-26

**Authors:** Gabriele Christa Krüger, Richard L. Jantz, Ericka N. L’Abbé

**Affiliations:** 1 Department of Anatomy, Faculty of Health Sciences, University of Pretoria, Private Bag X323, Arcadia, 0007 South Africa; 2 Department of Anthropology, University of Tennessee, Knoxville, TN USA

**Keywords:** Computed tomography, Population affinity, Indian South Africans, Cranial variation, Geometric morphometrics, Inter-landmark distances

## Abstract

Skeletal collections have provided the foundation for forensic anthropology in South Africa, offering invaluable reference material for developing and testing identification methods. However, these collections remain limited in both size and representativeness. Many are historically biased, restricted by donation practices, and unable to reflect the demographic and biological diversity of the country’s modern population. Indian South Africans, in particular, are notably absent due to cultural and religious beliefs that discourage body donation. This study explores the use of computed tomography (CT) as an alternative to overcome these limitations. A total of 408 high-resolution head CT scans representing Black, Coloured, Indian, and White South Africans were analysed using inter-landmark distances and geometric morphometrics. Measurement repeatability was high, with both intra- and inter-observer errors falling well within accepted forensic thresholds (< 2 mm; <3.5%). Significant population-level differences in cranial form were identified (*p* < 0.01). Black South Africans were consistently the most clearly differentiated group, while Coloured and Indian South Africans showed the greatest overlap, reflecting shared population affinity and historical admixture. Cross-validated classification accuracies ranged between 62% and 64%, indicating that both linear and shape-based analyses can capture relevant cranial variation. These results confirm that CT-derived data are reliable for examining cranial variation within contemporary South African samples and provide access to larger and more diverse datasets than traditional skeletal collections. This study establishes the first cranial reference data incorporating Indian South Africans and demonstrates how CT scanning can support the development of inclusive, population-specific standards for forensic anthropology in South Africa.

##  Introduction

 Skeletal collections have long served as the foundation for forensic anthropology, providing the comparative material needed to develop and to validate methods for estimating biological profiles in unidentified individuals. In South Africa, collections such as the Raymond A. Dart, Pretoria Bone, and Kirsten Collections have been essential in establishing standards for sex and population affinity estimation [1–3]. However, these valuable resources are not without limitations. They remain historically biased toward certain demographic groups, are constrained in size by donation rates and storage capacity, and, being static by nature, fail to reflect the evolving diversity of the modern South African population. In particular, Indian South Africans are almost entirely absent. This has been linked to cultural and religious traditions that emphasise the respectful and timely handling of the deceased, often favouring cremation and discouraging post-mortem procedures or long-term retention of human remains for research purposes [4]. These limitations have direct implications for the accuracy and applicability of reference data used in population affinity estimation during forensic casework in the country.

 Computed tomography (CT) scanning offers a practical alternative that can mitigate some of the ethical and logistical challenges associated with traditional skeletal collections. CT-derived three-dimensional (3D) cranial models are compatible with a range of analytical approaches including traditional osteometrics, inter-landmark distances (ILDs), and geometric morphometrics (GM), thereby allowing for comprehensive assessment of cranial size and shape variation [5–7]. Importantly, CT-based measurements have been shown to be accurate and repeatable within acceptable error margins when compared with data obtained from dry skeletal material [8–10]. As such, CT imaging may facilitate the development of larger and more contemporary population-relevant reference material.

 Population affinity estimation remains an important component of the biological profile, yet existing South African standards do not adequately reflect the diversity of the population. Traditional morphoscopic approaches, such as Walker’s [11] cranial scoring system, are still widely used, but their performance declines when applied outside the populations from which they were originally derived, with classification rates dropping to as low as 31% in South African samples [12]. Osteometric approaches implemented through programs such as Fordisc 3.1 offer more statistically robust frameworks; however, they are similarly limited when applied to admixed or underrepresented groups [13–18].

 Importantly, population affinity estimation in forensic anthropology is inherently probabilistic rather than deterministic. Human biological variation exists along continua, and morphological overlap between groups is expected, particularly in historically admixed populations such as those present in South Africa. Accordingly, the value of population affinity assessment does not lie in definitive classification, but in its ability to contribute probabilistic contextual information that may assist in narrowing the pool of possible matches during the identification process. The development of contemporary, population-relevant reference datasets is therefore essential to ensure that such assessments reflect present-day variation rather than historical skeletal collections that may no longer represent the individuals encountered in modern medico-legal contexts.

 Within this context, methodological approaches that are able to model variation as continuous rather than discrete are particularly relevant. Integrating CT-derived 3D cranial data with a GM approach offers one possible way forward. By modelling variation as continuous rather than discrete, these methods allow for a more nuanced exploration of cranial diversity while reducing reliance on typological assumptions.

 In South Africa, where identification is often complicated by incomplete documentation, migration, and individuals who may not be formally recorded in national systems, biological indicators derived from skeletal analysis may provide important contextual information when other avenues are limited. The country’s population structure reflects a complex history of migration, admixture, and social classification. Individuals who self-identify as Indian or Asian constitute a relatively small proportion of the national population (approximately 2.7%) [19], yet unidentified remains encountered in medico-legal contexts must still be assessed within frameworks that reflect the full diversity of the contemporary population. In practice, individuals may not always be readily assignable to socially recognised categories, further complicating identification efforts and highlighting the need for biological approaches that engage with present-day variation rather than historically derived reference samples.

 Advances in medical imaging provide an opportunity to generate modern comparative samples using CT scans obtained from living or recently deceased individuals. Access to such material allows for the examination of cranial morphological variation within populations that more closely reflect those encountered in current medico-legal contexts. CT-derived cranial models offer a non-destructive means of capturing three-dimensional anatomical detail while enabling the inclusion of individuals who would otherwise be absent from traditional skeletal collections. In this way, imaging-based datasets may contribute to the development of updated reference frameworks grounded in present-day biological diversity.

 The present study uses CT-derived cranial models to investigate population variation among contemporary South Africans, with particular emphasis on the underrepresented Indian South African population group. Cranial variation across Black, Coloured, Indian, and White South Africans was assessed using linear measurements, ILDs, and GM to evaluate their utility in modelling population-level cranial variation within a contemporary South African CT-derived sample. In doing so, this research highlights the potential of CT-based datasets to address longstanding limitations associated with skeletal collections and to provide contemporary comparative data that may inform future population-specific forensic research in South Africa.

## Materials and methods

 The study sample comprised 408 head CT scans representing Black, Coloured, White, and Indian South Africans, with equal numbers of males and females in each group (n = 51 per group). Only adults (≥18 years) were included to ensure complete cranial growth and development and individuals were selected according to the inclusion criteria and balanced to obtain equal representation of sex and population groups. Scans were retrospectively collected using convenience sampling from available hospital archives at three major South African hospitals: Steve Biko Academic Hospital (Pretoria, Gauteng), Groote Schuur Hospital (Cape Town, Western Cape), and Inkosi Albert Luthuli Central Hospital (Durban, KwaZulu-Natal). These sites were selected to reflect regional population distributions, with Coloured individuals sampled primarily from the Western Cape, and Indian individuals primarily from KwaZulu-Natal, while Black and White South Africans were sampled across all three regions. Ethical approval for the research was obtained through the University of Pretoria Research Ethics Committee (771/2018), and approval for access to the scans was approved through the National Health Research Database (KZ_201901_011 and WC_201901_014).

 Only scans with a slice thickness below 0.625 mm were included to ensure isotropy and high-resolution reconstructions [20–22]. Scans showing pathology, trauma, or artefacts affecting cranial landmarks were excluded. Age distributions were not matched between groups, as the primary aim was to obtain sufficient numbers of adult individuals meeting the imaging quality and demographic inclusion criteria.

 Hospitals did not consistently record population affinity in patient metadata. In cases where this information was unavailable, population affinity was cautiously inferred using recorded names and surnames. Only cases in which names were strongly and consistently associated with a particular population group were retained, and where any uncertainty existed, the scan was excluded from the sample. This approach has precedent in retrospective research where self-reported ethnicity is unavailable, with surname-based inference commonly used as a pragmatic proxy in epidemiological and population-based studies [23–25]. In the South African context, where historically structured population boundaries and relatively high levels of endogamy persist, this method may offer a cautious but workable means of approximating population affinity in the absence of recorded demographic data. Nevertheless, it does not constitute definitive classification and should be interpreted as an informed estimate, representing a limitation of the sample.

 All data were anonymised, with only sex, age, and estimated population affinity retained for analysis.

 Each scan was aligned to the Frankfurt Horizontal Plane using Avizo 9 (Thermo Scientific™; license: AVIZO.59228), with bilateral porion and inferior orbital margin landmarks used for alignment [26]. Cranial segmentation and volume rendering were performed using the Treatment and Increased Vision for Medical Imaging (TIVMI) software. This software applies a modified half-maximum height (HMH) thresholding protocol, which has been shown to reduce error rates compared to alternative segmentation approaches [27–30]. The resulting 3D cranial models formed the basis for all subsequent landmark placement and measurement.

### Landmark collection and measurements

**Fig. 1:**
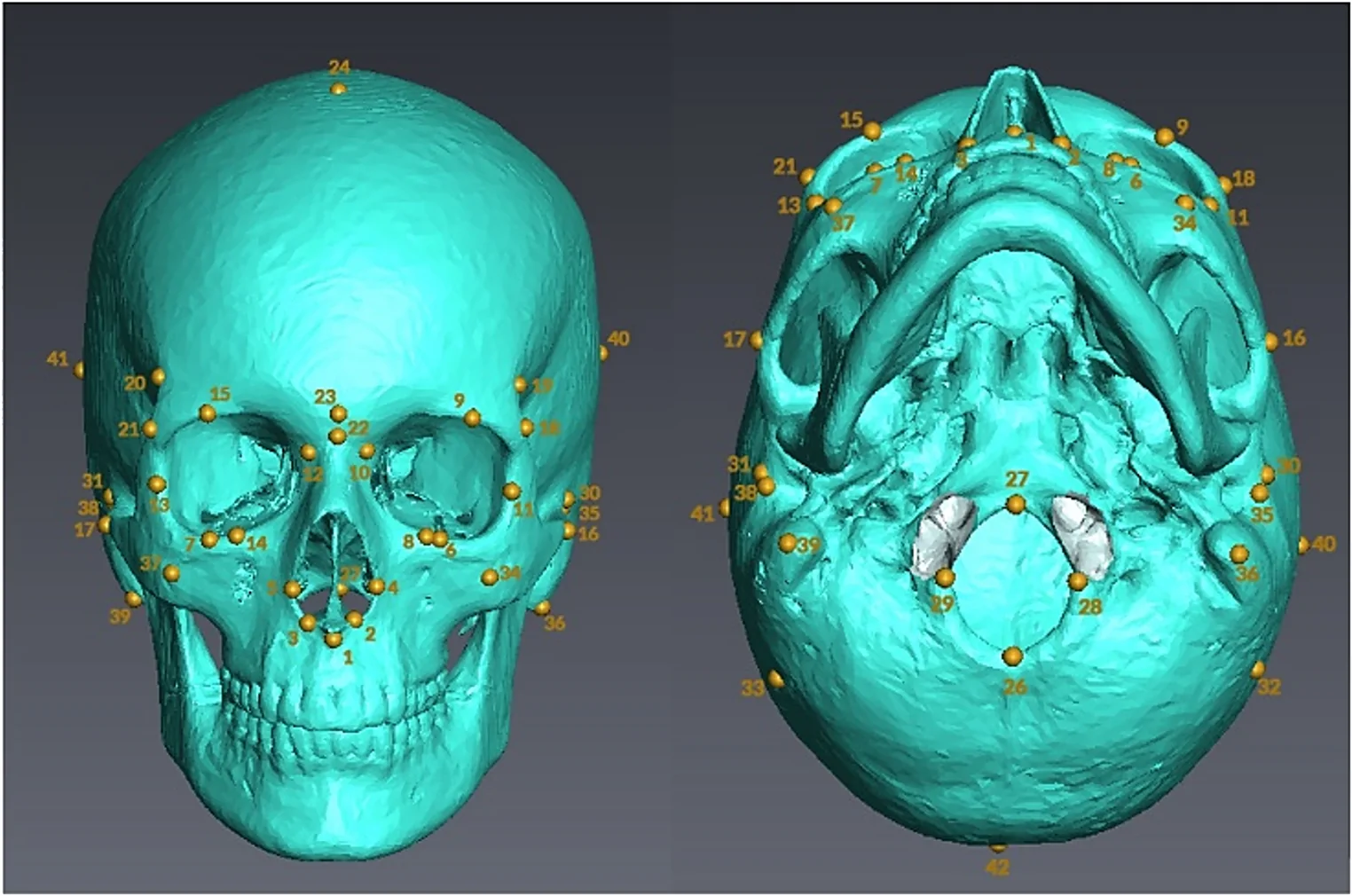
Landmarks on the anterior and inferior views of the skull

 A total of 42 landmarks were digitised on each cranium, comprising 40 standard cranial landmarks [31] and an additional bilateral mid-zygomaticomaxillary suture landmark to capture malar projection. Three-dimensional landmark coordinates (x, y, z) were exported for analysis. Inter-landmark distances (ILDs), incorporating both established anthropometric variables [32] and exploratory measures (Table 1; Fig. 1) [7], were calculated as the Euclidean distance between pairs of landmarks according to the formula:

$$ILD=\surd\left[\left(x_2-x_1\right)^2+\left(y_2-y_1\right)^2+\left(z_2-z_1\right)^2\right]$$

where (x_1_), (y_1_), and (z_1_) represent the coordinates of the first landmark and (x_2_), (y_2_), and (z_2_) represent the coordinates of the second landmark. Landmark coordinates were also exported for geometric morphometric analyses conducted in MorphoJ [5].

**Table 1:** Inter-landmark distance abbreviations and landmarks involved

Abbreviation	Landmark 1	Landmark number	Landmark 2	Landmark number
GOL	glabella	23	opisthocranion	42
XCB	left eurion	40	right eurion	41
ZYB	left zygion	16	right zygion	17
BBH	basion	27	bregma	24
BNL	basion	27	nasion	22
ASB	left asterion	32	right asterion	33
WFB	left frontotemporale	19	right frontotemporale	20
UFBR	left frontomalare temporale	18	right frontomalare temporale	21
NLHL	nasion	22	left most inferior nasal border	2
NLHR	nasion	22	right most inferior nasal border	3
NLH	average of NLHL and NLHR			
NLB	left alare	4	right alare	5
OBBL	left dacryon	10	left ectoconchion	11
OBBR	right dacryon	12	right ectoconchion	13
OBHL	left lower orbital border	8	left upper orbital border	9
OBHR	right lower orbital border	14	right upper orbital border	15
EKB	left ectoconchion	11	right ectoconchion	13
DKB	left dacryon	10	right dacryon	12
FRC	nasion	22	bregma	24
PAC	bregma	24	lambda	25
OCC	lambda	25	opisthion	26
FOL	opisthion	26	basion	27
FOB	left border of foramen magnum (FOB point)	28	right border of foramen magnum (FOB point)	29
MDHL	left porion	35	left mastoidale	36
MDHR	right porion	38	right mastoidale	39
AUB	left radiculare	30	right radiculare	31
NOL	nasion	22	opisthocranion	42
MOW	left zygoorbitale	6	right zygoorbitale	7
NLH2	nasion	22	supspinale	1
MAPL	left mid-malar projection point	34	left porion	35
MAPR	right mid-malar projection point	37	right porion	38
MAP2L	left mid-malar projection point	34	left ectoconchion	11
MAP2R	right mid-malar projection point	37	right ectoconchion	13
MAP3L	left mid-malar projection point	34	left asterion	32
MAP3R	right mid-malar projection point	37	right asterion	33

###  Repeatability

 Repeatability of landmark placement and derived measurements was evaluated using absolute and relative technical error of measurement (TEM and %TEM). Acceptable thresholds were set at <2 mm and <3.5%, respectively [17, 33, 34]. For intra-observer error assessment, landmarks were placed twice by the principal investigator on a subset of 15 crania, with a 14-day interval between landmarking sessions. For inter-observer error assessment, the same 15 crania were independently landmarked once by a second observer trained in cranial landmark identification using a standardised protocol. Bland-Altman plots were generated in R [35] to visualise intra- and inter-observer agreement [36].

 For GM analyses, dispersion error was additionally calculated to assess landmark repeatability. Mean Euclidean distance was computed for each landmark across repeated placements by both observers.

### Statistical analyses

 Descriptive statistics (means and standard deviations, and variance) were calculated for all variables. Differences between sex and population groups were assessed using analysis of variance (ANOVA) with Tukey’s HSD *post hoc* tests [37, 38].

 Linear discriminant analysis (LDA) with leave-one-out cross-validation (LOOCV) was performed using FD3 software to evaluate classification accuracy, with forward stepwise variable selection employed to optimise model performance [14, 39].

 For GM analyses, landmark coordinates underwent Generalised Procrustes Analysis (GPA) to remove variation related to size, orientation, and position [40, 41]. Shape variation was explored using principal component analysis (PCA) [42], Procrustes ANOVA with 1000 permutations and ordinary least squares [43], and 50-50 MANOVA (multivariate ANOVA) [44]. Canonical variate analysis (CVA) was conducted in MorphoJ and R to assess the discriminatory potential of shape variables [45].

## Results

### Inter-landmark distances (ILDs)

#### Repeatability

 Repeatability tests confirmed that measurement error was within acceptable limits. The mean intra-observer TEM was 0.48 mm (0.11–1.26 mm) with a mean%TEM of 1.02%, while inter-observer TEM averaged 0.73 mm (0.17–2.11 mm) with a mean %TEM of 1.45% (Table 2). Only a small number of landmarks exceeded established thresholds (<2 mm; <3.5%), and in each case the excess was negligible (<0.13 mm).

**Table 2:** Absolute (TEM) and relative technical error of measurement (%TEM) for the 35 ILDs. Bold indicates values greater than the generally acceptable standards for intra- and inter-observer error for ILDs in anthropological analyses

ILD	Intra-observer error		Inter-observer error	
	TEM (in mm)	%TEM (in %)	TEM (in mm)	%TEM (in %)
GOL	0.18	0.10	0.36	0.19
XCB	0.39	0.28	0.18	0.13
ZYB	0.18	0.13	0.48	0.37
BBH	0.16	0.11	0.17	0.12
BNL	0.33	0.32	0.85	0.82
ASB	0.46	0.41	0.50	0.45
WFB	0.11	0.11	0.30	0.31
UFBR	0.17	0.16	0.46	0.43
NLHL	0.35	0.68	0.44	0.86
NLHR	0.37	0.71	0.62	1.21
NLH	0.34	0.66	0.49	0.94
NLB	0.20	0.73	0.45	1.64
OBBL	0.78	1.78	0.86	1.93
OBBR	0.40	0.91	0.67	1.51
OBHL	0.21	0.58	0.60	1.63
OBHR	0.37	1.00	0.58	1.57
EKB	0.62	0.63	1.70	1.71
DKB	0.65	3.09	0.68	3.29
FRC	0.30	0.26	0.42	0.37
PAC	0.62	0.52	0.71	0.60
OCC	0.30	0.30	0.47	0.47
FOL	0.29	0.74	1.13	2.81
FOB	0.45	1.48	0.95	3.09
MDHL	0.55	1.67	0.70	2.14
MDHR	0.31	0.93	0.74	2.26
AUB	0.25	0.21	0.42	0.34
NOL	0.22	0.12	0.49	0.26
MOW	1.21	2.22	**2.11**	**3.88**
NLH2	0.42	0.78	0.78	1.44
MAPL	0.83	1.09	1.12	1.48
MAPR	0.87	1.11	0.89	1.14
MAP2L	1.05	**4.95**	1.01	**4.59**
MAP2R	1.26	**5.49**	1.20	**5.26**
MAP3L	0.90	0.71	1.01	0.80
MAP3R	0.76	0.60	1.02	0.81

**Fig. 2:**
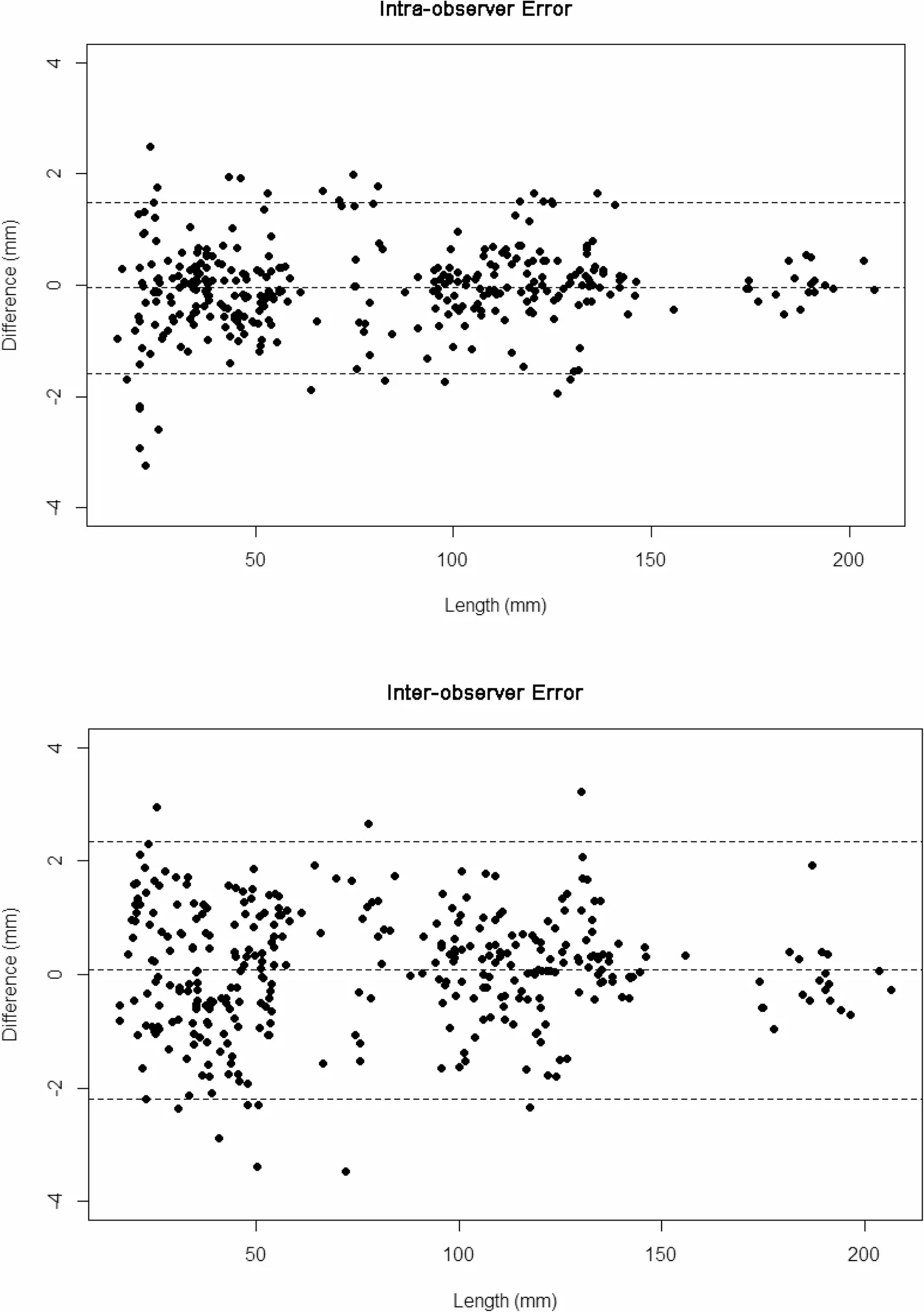
Bland-Altman plot illustrating the intra-observer agreement (top) when the principle investigator repeated the landmark placement and inter-observer agreement (bottom) when a second observer repeated the landmark placement on 15 randomly selected 3D cranial models. The majority of the resulting ILDs fall within the upper and lower agreement levels (dashed lines), which are based on the SD

 Mid-orbital width (MOW) and the bilateral mid-malar projection to ectoconchion distances (MAP2L and MAP2R) displayed comparatively higher error rates and were excluded from further analyses. Bland-Altman plots demonstrated high agreement and minimal systematic bias, with slightly greater dispersion observed in inter-observer comparisons (Fig. 2).

#### Descriptive statistics and group differences

 Descriptive statistics indicated clear population-level trends in cranial size (Table 3). White South Africans tended to exhibit the largest cranial vault and vertical dimensions, while Black South Africans displayed the greatest cranial lengths and midfacial breadths. Indian South Africans consistently demonstrated the smallest overall ILD means, and Coloured South Africans were generally intermediate across most measurements.

**Table 3:** Black, Coloured, Indian and White South African group means and SD for the various ILDs. Bold indicates the largest mean for each ILD

ILD	Black South Africans			Coloured South Africans			Indian South Africans			White South Africans		
	n*	mean	SD	n*	mean	SD	n*	mean	SD	n*	mean	SD
GOL	101	**185.1**	7.5	101	182.0	7.5	102	180.0	8.0	102	184.9	8.2
XCB	98	136.6	4.7	101	136.0	5.4	102	134.1	5.5	102	**140.5**	5.4
ZYB	101	**127.2**	5.9	101	123.9	5.0	101	123.1	4.9	101	126.0	5.6
BBH	101	134.2	5.6	100	134.0	5.6	100	131.3	5.9	102	**138.0**	6.0
BNL	102	101.3	5.1	100	101.4	5.0	102	101.3	5.6	102	**104.5**	5.5
ASB	102	109.4	5.4	100	108.9	4.6	101	106.8	5.8	102	**113.3**	5.5
WFB	102	**95.6**	5.2	102	94.0	4.3	102	92.9	4.4	101	95.0	4.5
UFBR	102	**105.4**	4.9	101	102.5	4.0	101	101.3	4.1	97	102.4	4.4
NLHL	101	49.5	3.0	101	51.3	3.4	102	50.8	4.1	102	**53.2**	3.4
NLHR	102	49.6	3.0	102	51.4	3.5	102	50.8	4.0	101	**53.1**	3.2
NLH	100	49.5	2.8	102	51.4	3.5	102	50.8	4.0	101	**53.2**	3.2
NLB	99	**27.9**	2.0	98	25.3	1.5	100	24.7	1.7	102	25.1	2.0
OBBL	102	44.2	2.5	99	44.4	2.1	96	**44.7**	1.8	100	44.1	2.1
OBBR	99	44.5	2.3	100	44.1	2.1	99	**44.7**	1.8	102	44.3	2.2
OBHL	101	35.4	1.9	101	**35.9**	2.0	101	35.2	1.8	101	35.8	2.0
OBHR	100	35.7	1.9	102	**35.7**	2.1	101	35.1	1.9	101	35.5	2.0
EKB	100	**99.5**	4.0	102	96.7	4.0	98	95.9	3.4	98	96.9	3.4
DKB	100	**19.5**	2.4	99	17.1	2.0	101	16.4	1.9	102	17.7	1.7
FRC	100	112.4	5.5	101	111.5	4.6	101	110.5	5.4	102	**114.0**	5.4
PAC	101	**116.5**	6.9	102	114.2	7.3	100	112.1	6.1	102	115.1	7.4
OCC	101	98.2	5.2	102	95.1	6.1	102	94.5	5.1	102	**100.2**	5.3
FOL	101	37.6	2.5	102	38.0	2.6	99	36.5	2.4	101	**38.1**	2.7
FOB	102	30.4	2.4	101	31.6	2.2	101	30.6	2.2	98	**32.4**	2.3
MDHL	102	31.8	3.8	102	30.7	3.6	99	29.9	3.1	99	**31.9**	3.2
MDHR	102	32.0	3.9	102	31.1	3.4	99	30.8	3.5	102	**32.7**	3.4
AUB	100	118.3	4.9	100	118.2	5.0	98	116.5	4.2	102	**121.5**	5.3
NOL	101	**183.2**	7.3	100	179.7	7.2	102	178.0	7.7	100	182.3	7.1
MAPL	97	**74.8**	3.8	102	72.9	3.9	101	71.2	4.3	99	73.2	4.1
MAPR	101	**75.4**	4.3	102	73.1	4.1	101	71.5	3.9	102	73.7	4.1
MAP3L	101	**123.4**	6.1	100	120.2	5.4	100	118.1	6.3	101	121.9	7.0
MAP3R	100	**124.5**	6.0	99	121.5	5.5	100	118.2	6.8	101	122.2	7.2
NLH2	102	52.9	3.3	102	55.4	3.8	101	54.7	4.0	99	**57.1**	3.2

* Sample sizes vary slightly between inter-landmark distances due to occasional missing landmark data resulting from image quality limitations or difficulties in landmark identification on some CT reconstructions

 Sexual dimorphism was evident across all populations, with males exhibiting larger mean ILDs than females for all variables, with the exception of DKB in Black South Africans (Tables 4, 5, 6 and 7). White and Black South Africans generally demonstrated the largest ILD means across many variables, whereas Indian South Africans consistently exhibited the smallest overall means, with Coloured South Africans typically showing intermediate values. Sex-specific descriptive statistics are provided in Table 8. These observed patterns were explored further through inferential statistical testing.

**Table 4:** Black South African male and female means and SD for the various ILDs. Bold indicates the larger mean for each ILD

ILD	Black SA females			Black SA males			ILD	Black SA females			Black SA males		
	*n*	mean	SD	*n*	mean	SD		*n*	mean	SD	*n*	mean	SD
GOL	50	181.3	6.0	51	**188.8**	7.0	EKB	50	98.0	3.5	50	**101.0**	4.0
XCB	49	134.8	4.0	49	**138.4**	4.7	DKB	49	**19.8**	2.4	51	19.2	2.4
ZYB	51	124.0	4.5	50	**130.5**	5.3	FRC	51	110.5	5.2	49	**114.3**	5.1
BBH	51	131.8	4.9	50	**136.6**	5.3	PAC	50	115.0	7.0	51	**117.9**	6.5
BNL	51	98.2	4.1	51	**104.4**	4.1	OCC	50	97.9	5.1	51	**98.6**	5.3
ASB	51	108.3	5.4	51	**110.5**	5.3	FOL	51	36.6	2.0	50	**38.6**	2.6
WFB	51	94.3	4.6	51	**96.8**	5.6	FOB	51	29.4	2.3	51	**31.4**	2.0
UFBR	51	103.6	4.3	51	**107.3**	4.7	MDHL	51	29.7	3.2	51	**33.9**	3.2
NLHL	51	48.0	2.7	50	**51.1**	2.4	MDHR	51	29.9	3.2	51	**34.0**	3.4
NLHR	51	48.1	2.7	51	**51.2**	2.5	AUB	51	116.6	4.8	49	**120.1**	4.5
NLH	51	48.0	2.7	49	**50.9**	2.2	NOL	50	179.7	5.9	51	**186.7**	6.9
NLB	49	27.9	2.0	50	**28.0**	2.0	MAPL	49	73.1	3.5	48	**76.5**	3.2
OBBL	51	43.1	2.0	51	**45.3**	2.6	MAPR	51	74.0	4.0	50	**76.9**	4.1
OBBR	49	43.6	1.7	50	**45.3**	2.5	MAP3L	50	120.9	5.6	51	**125.8**	5.7
OBHL	51	35.3	1.9	50	**35.6**	2.0	MAP3R	49	122.0	5.4	51	**127.0**	5.5
OBHR	49	35.6	1.8	51	**35.9**	2.1	NLH2	51	51.1	2.7	51	**54.7**	2.7

**Table 5:** Coloured South African male and female means and SD for the various ILDs. Bold indicates the larger mean for each ILD

ILD	Coloured SA females			Coloured SA males			ILD	Coloured SA females			Coloured SA males		
	*n*	mean	SD	*n*	mean	SD		*n*	mean	SD	*n*	mean	SD
GOL	50	178.3	5.9	51	**185.5**	7.2	EKB	51	95.6	3.4	51	**97.8**	4.2
XCB	51	133.5	4.6	50	**138.6**	5.1	DKB	49	17.0	2.0	50	**17.1**	2.1
ZYB	51	121.1	4.2	50	**126.8**	3.9	FRC	51	109.8	3.9	50	**113.3**	4.6
BBH	51	131.3	4.9	49	**136.8**	4.9	PAC	51	111.4	6.1	51	**117.1**	7.3
BNL	51	99.0	4.2	49	**103.9**	4.5	OCC	51	94.0	5.6	51	**96.3**	6.3
ASB	51	107.6	3.8	49	**110.3**	5.0	FOL	51	37.0	2.4	51	**38.9**	2.5
WFB	51	92.9	3.4	51	**95.2**	4.8	FOB	50	30.9	2.1	51	**32.4**	2.1
UFBR	51	101.7	3.4	50	**103.2**	4.5	MDHL	51	29.1	2.5	51	**32.3**	3.8
NLHL	51	49.9	2.7	50	**52.8**	3.5	MDHR	51	29.5	2.6	51	**32.7**	3.4
NLHR	51	49.8	2.6	51	**52.9**	3.6	AUB	50	115.6	4.3	50	**120.8**	4.4
NLH	51	49.8	2.6	51	**52.9**	3.6	NOL	50	176.5	6.2	50	**182.9**	6.6
NLB	50	25.3	1.4	48	**25.4**	1.7	MAPL	51	71.4	3.8	51	**74.4**	3.4
OBBL	50	43.8	1.9	49	**45.0**	2.1	MAPR	51	71.6	3.9	51	**74.7**	3.6
OBBR	50	43.7	1.9	50	**44.6**	2.1	MAP3L	50	117.7	4.5	50	**122.7**	5.0
OBHL	51	35.7	2.0	50	**36.0**	2.1	MAP3R	49	119.0	4.4	50	**124.0**	5.4
OBHR	51	35.4	2.0	51	**36.0**	2.2	NLH2	51	53.6	2.8	51	**57.1**	3.9

**Table 6:** Indian South African male and female means and SD for the various ILDs. Bold indicates the larger mean for each ILD

ILD	Indian SA females			Indian SA males			ILD	Indian SA females			Indian SA males		
	*n*	mean	SD	*n*	mean	SD		*n*	mean	SD	*n*	mean	SD
GOL	51	174.7	5.7	51	**185.3**	6.4	EKB	51	94.3	2.9	47	**97.7**	3.1
XCB	51	132.1	4.6	51	**136.2**	5.6	DKB	51	15.9	2.2	50	**16.8**	1.5
ZYB	50	120.3	3.8	51	**125.9**	4.2	FRC	51	107.5	4.3	50	**113.6**	4.6
BBH	50	127.5	4.0	50	**135.2**	4.8	PAC	49	109.1	5.4	51	**115.0**	5.3
BNL	51	97.5	3.4	51	**105.2**	4.7	OCC	51	93.1	4.9	51	**95.8**	4.9
ASB	51	105.4	4.8	50	**108.4**	6.4	FOL	51	35.6	2.4	48	**37.6**	1.9
WFB	51	90.7	3.6	51	**95.0**	4.0	FOB	51	29.8	1.8	50	**31.5**	2.2
UFBR	51	99.1	3.4	50	**103.5**	3.6	MDHL	51	28.0	2.2	48	**31.9**	2.7
NLHL	51	48.2	2.9	51	**53.5**	3.2	MDHR	51	28.6	2.4	48	**33.2**	2.8
NLHR	51	48.2	3.3	51	**53.5**	2.9	AUB	51	114.3	3.4	47	**118.8**	3.8
NLH	51	48.2	3.0	51	**53.5**	3.0	NOL	51	173.0	5.5	51	**182.9**	6.4
NLB	51	24.4	1.5	49	**25.0**	1.7	MAPL	51	68.9	3.2	50	**73.5**	4.0
OBBL	48	44.1	1.7	48	**45.3**	1.7	MAPR	51	69.8	2.9	50	**73.3**	4.0
OBBR	51	44.2	1.6	48	**45.1**	1.8	MAP3L	51	114.6	4.1	49	**121.7**	6.2
OBHL	50	34.8	1.7	51	**35.7**	1.9	MAP3R	51	114.3	4.8	49	**122.3**	6.2
OBHR	51	34.5	1.9	50	**35.7**	1.8	NLH2	51	52.0	2.8	50	**57.5**	3.1

**Table 7:** White South African male and female means and SD for the various ILDs. Bold indicates the larger mean for each ILD

ILD	White SA females			White SA males			ILD	White SA females			White SA males		
	*n*	mean	SD	*n*	mean	SD		*n*	mean	SD	*n*	mean	SD
GOL	51	179.5	5.5	51	**190.3**	6.8	EKB	51	95.1	2.7	47	**98.9**	3.0
XCB	51	137.5	4.4	51	**143.5**	4.5	DKB	51	17.7	1.3	51	**17.8**	2.0
ZYB	51	121.8	3.3	50	**130.3**	3.9	FRC	51	110.8	4.8	51	**117.2**	3.9
BBH	51	134.1	4.4	51	**141.9**	4.8	PAC	51	112.2	7.3	51	**118.0**	6.3
BNL	51	100.9	3.9	51	**108.1**	4.4	OCC	51	98.9	5.5	51	**101.5**	4.9
ASB	51	110.9	4.0	51	**115.6**	5.9	FOL	51	36.8	2.3	50	**39.4**	2.4
WFB	51	93.3	4.0	50	**96.7**	4.4	FOB	50	31.2	2.0	48	**33.6**	2.0
UFBR	49	99.9	3.0	48	**105.0**	4.1	MDHL	50	30.1	2.7	49	**33.7**	2.6
NLHL	51	51.8	2.9	51	**54.5**	3.4	MDHR	51	31.0	2.8	51	**34.3**	3.1
NLHR	50	51.9	2.7	51	**54.3**	3.1	AUB	51	118.2	3.8	51	**124.7**	4.5
NLH	50	51.9	2.7	51	**54.4**	3.2	NOL	51	178.0	5.5	49	**186.8**	5.8
NLB	51	24.8	1.9	51	**25.4**	2.0	MAPL	51	71.3	3.3	48	**75.3**	4.0
OBBL	51	42.7	1.6	49	**45.5**	1.6	MAPR	51	71.7	2.8	51	**75.7**	4.2
OBBR	51	42.9	1.9	51	**45.7**	1.6	MAP3L	51	117.2	4.5	50	**126.8**	5.6
OBHL	50	35.5	2.1	51	**36.0**	2.0	MAP3R	51	117.2	4.4	50	**127.3**	5.8
OBHR	51	35.3	1.9	50	**35.7**	2.1	NLH2	50	55.9	2.4	49	**58.3**	3.4

**Table 8:** Sex-specific descriptive statistics for cranial ILDs among Black, Coloured, Indian, and White South Africans. Bold indicates the larger mean for each ILD when separated by sex

ILD	Black SA females		Coloured SA females		Indian SA females		White SA females		Black SA males		Coloured SA males		Indian SA males		White SA males	
	mean	SD	mean	SD	mean	SD	mean	SD	mean	SD	mean	SD	mean	SD	mean	SD
GOL	**181.3**	6.0	178.3	5.9	174.7	5.7	179.5	5.5	188.8	7.0	185.5	7.2	185.3	6.4	**190.3**	6.8
XCB	134.8	4.0	133.5	4.6	132.1	4.6	**137.5**	4.4	138.4	4.7	138.6	5.1	136.2	5.6	**143.5**	4.5
ZYB	**124.0**	4.5	121.1	4.2	120.3	3.8	121.8	3.3	**130.5**	5.3	126.8	3.9	125.9	4.2	130.3	3.9
BBH	131.8	4.9	131.3	4.9	127.5	4.0	**134.1**	4.4	136.6	5.3	136.8	4.9	135.2	4.8	**141.9**	4.8
BNL	98.2	4.1	99.0	4.2	97.5	3.4	**100.9**	3.9	104.4	4.1	103.9	4.5	105.2	4.7	**108.1**	4.4
ASB	108.3	5.4	107.6	3.8	105.4	4.8	**110.9**	4.0	110.5	5.3	110.3	5.0	108.4	6.4	**115.6**	5.9
WFB	**94.3**	4.6	92.9	3.4	90.7	3.6	93.3	4.0	**96.8**	5.6	95.2	4.8	95.0	4.0	96.7	4.4
UFBR	**103.6**	4.3	101.7	3.4	99.1	3.4	99.9	3.0	**107.3**	4.7	103.2	4.5	103.5	3.6	105.0	4.1
NLHL	48.0	2.7	49.9	2.7	48.2	2.9	**51.8**	2.9	51.1	2.4	52.8	3.5	53.5	3.2	**54.5**	3.4
NLHR	48.1	2.7	49.8	2.6	48.2	3.3	**51.9**	2.7	51.2	2.5	52.9	3.6	53.5	2.9	**54.3**	3.1
NLH	48.0	2.7	49.8	2.6	48.2	3.0	**51.9**	2.7	50.9	2.2	52.9	3.6	53.5	3.0	**54.4**	3.2
NLB	**27.9**	2.0	25.3	1.4	24.4	1.5	24.8	1.9	**28.0**	2.0	25.4	1.7	25.0	1.7	25.4	2.0
OBBL	43.1	2.0	43.8	1.9	**44.1**	1.7	42.7	1.6	45.3	2.6	45.0	2.1	45.3	1.7	**45.5**	1.6
OBBR	43.6	1.7	43.7	1.9	**44.2**	1.6	42.9	1.9	45.3	2.5	44.6	2.1	45.1	1.8	**45.7**	1.6
OBHL	35.3	1.9	**35.7**	2.0	34.8	1.7	35.5	2.1	35.6	2.0	**36.0**	2.1	35.7	1.9	**36.0**	2.0
OBHR	**35.6**	1.8	35.4	2.0	34.5	1.9	35.3	1.9	35.9	2.1	**36.0**	2.2	35.7	1.8	35.7	2.1
EKB	**98.0**	3.5	95.6	3.4	94.3	2.9	95.1	2.7	**101.0**	4.0	97.8	4.2	97.7	3.1	98.9	3.0
DKB	**19.8**	2.4	17.0	2.0	15.9	2.2	17.7	1.3	**19.2**	2.4	17.1	2.1	16.8	1.5	17.8	2.0
FRC	110.5	5.2	109.8	3.9	107.5	4.3	**110.8**	4.8	114.3	5.1	113.3	4.6	113.6	4.6	**117.2**	3.9
PAC	**115.0**	7.0	111.4	6.1	109.1	5.4	112.2	7.3	117.9	6.5	117.1	7.3	115.0	5.3	**118.0**	6.3
OCC	97.9	5.1	94.0	5.6	93.1	4.9	**98.9**	5.5	98.6	5.3	96.3	6.3	95.8	4.9	**101.5**	4.9
FOL	36.6	2.0	**37.0**	2.4	35.6	2.4	36.8	2.3	38.6	2.6	38.9	2.5	37.6	1.9	**39.4**	2.4
FOB	29.4	2.3	30.9	2.1	29.8	1.8	**31.2**	2.0	31.4	2.0	32.4	2.1	31.5	2.2	**33.6**	2.0
MDHL	29.7	3.2	29.1	2.5	28.0	2.2	**30.1**	2.7	**33.9**	3.2	32.3	3.8	31.9	2.7	33.7	2.6
MDHR	29.9	3.2	29.5	2.6	28.6	2.4	**31.0**	2.8	34.0	3.4	32.7	3.4	33.2	2.8	**34.3**	3.1
AUB	116.6	4.8	115.6	4.3	114.3	3.4	**118.2**	3.8	120.1	4.5	120.8	4.4	118.8	3.8	**124.7**	4.5
NOL	**179.7**	5.9	176.5	6.2	173.0	5.5	178.0	5.5	186.7	6.9	182.9	6.6	182.9	6.4	**186.8**	5.8
MAPL	**73.1**	3.5	71.4	3.8	68.9	3.2	71.3	3.3	**76.5**	3.2	74.4	3.4	73.5	4.0	75.3	4.0
MAPR	**74.0**	4.0	71.6	3.9	69.8	2.9	71.7	2.8	**76.9**	4.1	74.7	3.6	73.3	4.0	75.7	4.2
MAP3L	**120.9**	5.6	117.7	4.5	114.6	4.1	117.2	4.5	125.8	5.7	122.7	5.0	121.7	6.2	**126.8**	5.6
MAP3R	**122.0**	5.4	119.0	4.4	114.3	4.8	117.2	4.4	127.0	5.5	124.0	5.4	122.3	6.2	**127.3**	5.8
NLH2	51.1	2.7	53.6	2.8	52.0	2.8	**55.9**	2.4	54.7	2.7	57.1	3.9	57.5	3.1	**58.3**	3.4

 Analysis of variance (ANOVA) revealed significant differences between populations for the majority of ILDs, as well as significant sex effects for most variables (Table 8). Several orbital height and breadth measurements showed greater overlap among groups. Significant population-sex interactions were identified for multiple ILDs, supporting the use of sex-specific analyses. Tukey’s HSD *post hoc* tests confirmed overlap among at least two populations for every ILD, with the greatest overlap occurring between Coloured and Indian South Africans (Table 9).

**Table 9:** ANOVA to assess the ILD for significant differences between the sexes and populations, as well as for any significant interactions between sex and population affinity. A post hoc Tukey’s HSD test was used to explore which populations overlapped for the various ILDs. Bold p-values indicate non-significance

	ANOVA						Tukey’s HSD
ILD	Sex		Population Affinity		Pop*Sex interaction		Overlap between groups
	F-value	*p*-value	F-value	*p*-value	F-value	*p*-value	
GOL	182.9	< 0.001	10.1	< 0.001	2.3	**0.076**	W-B; I-C
XCB	81.2	< 0.001	26.1	< 0.001	1.3	**0.263**	C-B; I-C
ZYB	218.6	< 0.001	12.5	< 0.001	2.7	< 0.05	W-B; I-C
BBH	147.2	< 0.001	22.5	< 0.001	2.6	**0.050**	C-B
BNL	224.5	< 0.001	8.9	< 0.001	2.3	**0.074**	C-B; I-B; I-C
ASB	31.5	< 0.001	25.3	< 0.001	1.1	**0.345**	C-B
WFB	49.3	< 0.001	6.7	< 0.001	1.1	**0.332**	C-B; W-B; I-C; W-C
UFBR	75.4	< 0.001	16.7	< 0.001	3.9	< 0.01	I-C; W-C; W-I
NLHL	118.0	< 0.001	18.8	< 0.001	4.5	< 0.01	I-C
NLHR	118.7	< 0.001	17.5	< 0.001	4.3	< 0.01	I-C; I-B
NLH	118.8	< 0.001	20.3	< 0.001	4.8	< 0.01	I-C
NLB	2.8	**0.0929**	66.6	< 0.001	0.6	**0.619**	I-C; W-C; W-I
OBBL	88.6	< 0.001	1.6	**0.191**	3.9	< 0.01	All groups
OBBR	66.8	< 0.001	1.2	**0.307**	5.6	< 0.001	All groups
OBHL	7.2	< 0.01	2.2	**0.0839**	0.6	**0.637**	All groups
OBHR	10.6	< 0.01	2.2	**0.0932**	1.2	**0.304**	All groups
EKB	73.1	< 0.001	17.4	< 0.001	1.0	**0.399**	I-C; W-C; W-I
DKB	0.3	**0.598**	44.6	< 0.001	2.5	**0.062**	I-C; W-C
FRC	111.4	< 0.001	8.0	< 0.001	2.9	< 0.05	W-B; I-C; C-B; I-B
PAC	60.0	< 0.001	7.0	< 0.001	1.2	**0.300**	C-B; W-B; I-C; W-C
OCC	12.8	< 0.001	24.8	< 0.001	0.8	**0.508**	I-C; W-B
FOL	82.3	< 0.001	7.5	< 0.001	0.6	**0.604**	C-B; W-B; W-C
FOB	73.9	< 0.001	15.5	< 0.001	0.9	**0.435**	I-B; W-C
MDHL	154.2	< 0.001	7.7	< 0.001	0.7	**0.576**	C-B; W-B; I-C; W-C
MDHR	155.6	< 0.001	5.6	< 0.001	1.1	**0.341**	C-B; I-B; W-B; I-C
AUB	117.8	< 0.001	18.1	< 0.001	2.2	**0.084**	C-B; I-C
NOL	154.7	< 0.001	10.9	< 0.001	1.7	**0.163**	W-B; I-C; W-C
MAPL	97.6	< 0.001	13.6	< 0.001	1.0	**0.389**	W-C
MAPR	75.0	< 0.001	15.7	< 0.001	0.6	**0.651**	W-C
MAP3L	14.0	< 0.001	13.5	< 0.001	4.5	< 0.01	W-B; I-C; W-C
MAP3R	151.8	< 0.001	16.5	< 0.001	5.8	< 0.001	W-C; W-B
NLH2	124.9	< 0.001	23.3	< 0.001	4.3	< 0.01	I-C

#### Classification accuracy (ILDs)

**Fig. 3:**
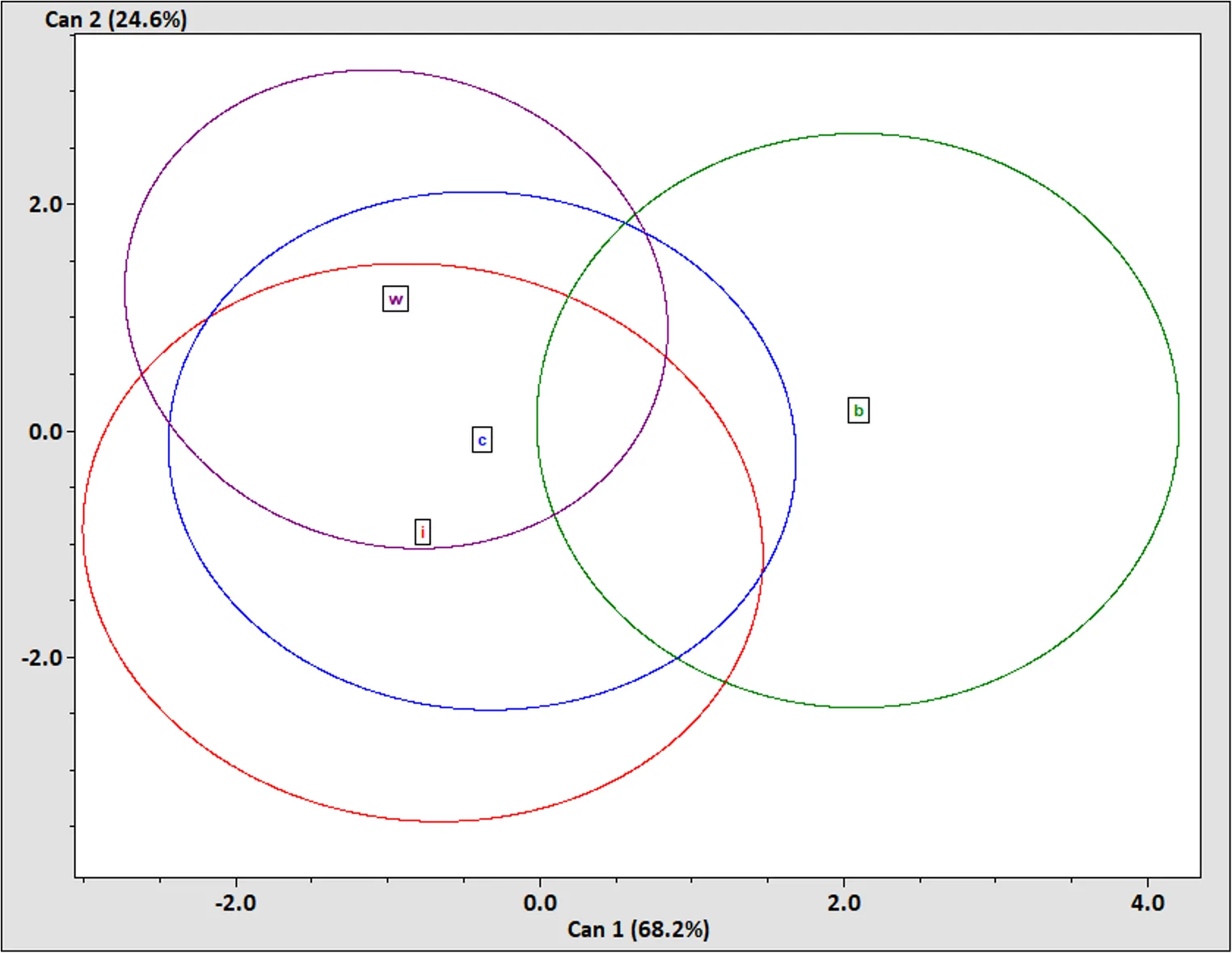
Graph illustrating the classification of the four population groups. The letters depict the group centroids. Note the extensive group overlap among Coloured, Indian and White South Africans

 Following the identification of significant population-level differences in cranial size, the ability of these variables to discriminate among groups was subsequently assessed through classification analyses. LDA with LOOCV produced an overall correct classification rate of 62.2% for population affinity estimation based on ILDs (Table 10). Black South Africans achieved the highest correct classification rates, whereas Coloured South Africans demonstrated the greatest degree of misclassification. Most misclassifications occurred between Coloured and Indian South Africans, with additional overlap involving White South Africans (Fig. 3).

**Table 10:** Classification matrix and cross-validated (CV) correct classification rates when estimating population affinity using 24 Forward Wilks selected cranial ILDs

From group	Into group				Total	Correct classification
	B	C	I	W		
**B**	63	10	0	4	77	81.8%
**C**	9	32	20	16	77	41.6%
**I**	5	15	44	12	76	57.9%
**W**	3	14	10	55	82	67.1%

Total correct = 194 of 312

CV correct classification = 62.2%

Mean Cohen’s kappa = 0.496

Mean Balanced Accuracy = 0.747

**Fig. 4:**
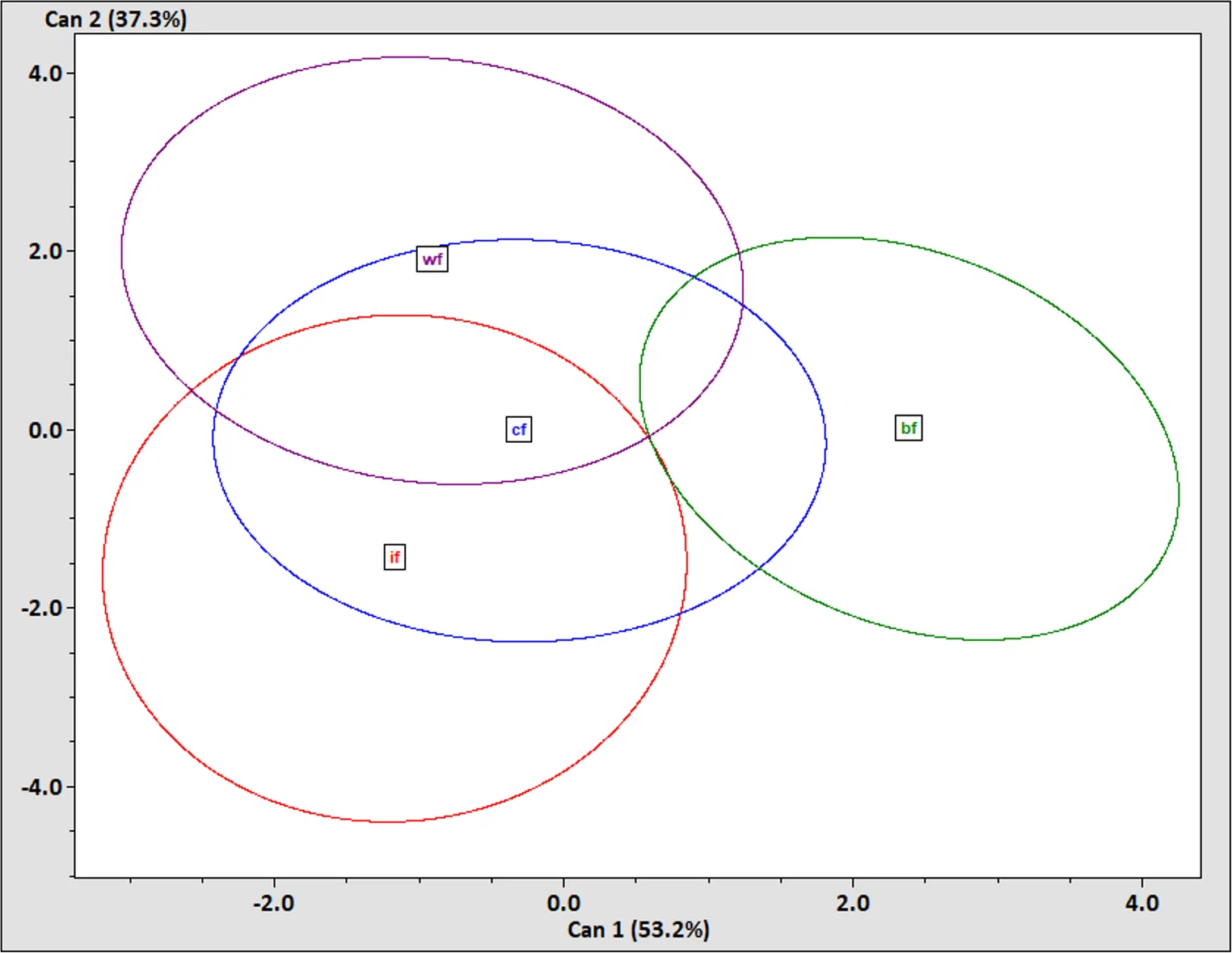
Graph illustrating the classification of the females of the four population groups. The letters depict the group centroids

**Fig. 5:**
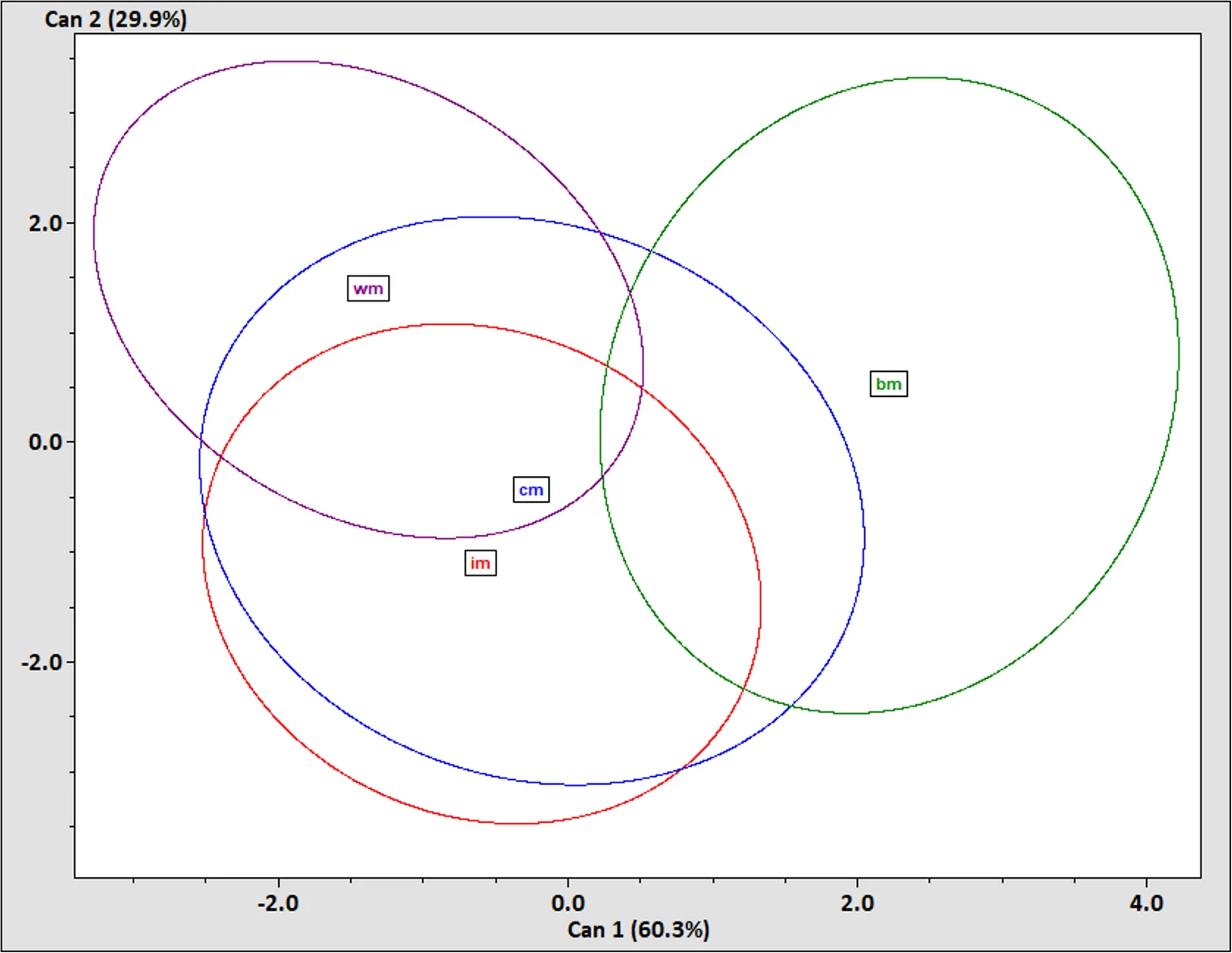
Graph illustrating the classification of the males of the four population groups. The letters depict the group centroids

 When sexes were analysed separately, classification accuracy improved overall. Females yielded higher correct classification rates than males, with Black South African females consistently the most accurately classified group and Coloured South African males the least accurately classified (Tables 11 and 12; Figs. 4 and 5). Despite improved separation in sex-specific analyses, substantial overlap remained, particularly between Coloured and Indian South Africans. To further explore these patterns independent of size effects, shape variation was assessed using geometric morphometric methods.

**Table 11:** Classification matrix and cross-validated (CV) correct classification rates when estimating population affinity among the females of the various population groups using 22 Forward Wilks selected cranial ILDs

From group	Into group				Total	Correct classification
	BF	CF	IF	WF		
**BF**	31	4	0	3	38	81.6%
**CF**	5	23	5	8	41	56.1%
**IF**	2	4	32	4	44	77.3%
**WF**	2	6	2	36	46	78.3%

Total correct = 124 of 169

CV correct classification = 73.4%

Mean Cohen’s kappa = 0.645

Mean Balanced Accuracy = 0.822

**Table 12:** Classification matrix and cross-validated (CV) correct classification rates when estimating population affinity among the males of the various population groups using 26 Forward Wilks selected cranial ILDs

From group	Into group				Total	Correct classification
	BM	CM	IM	WM		
**BM**	25	9	2	2	38	65.8%
**CM**	4	15	11	7	37	40.5%
**IM**	1	13	15	5	34	44.1%
**WM**	1	6	5	28	40	70.0%

Total correct = 83 of 149

CV correct classification = 55.7%

Mean Cohen’s kappa = 0.411

Mean Balanced Accuracy = 0.702

### Geometric morphometrics (GM)

#### Repeatability and Shape Variation

**Fig. 6:**
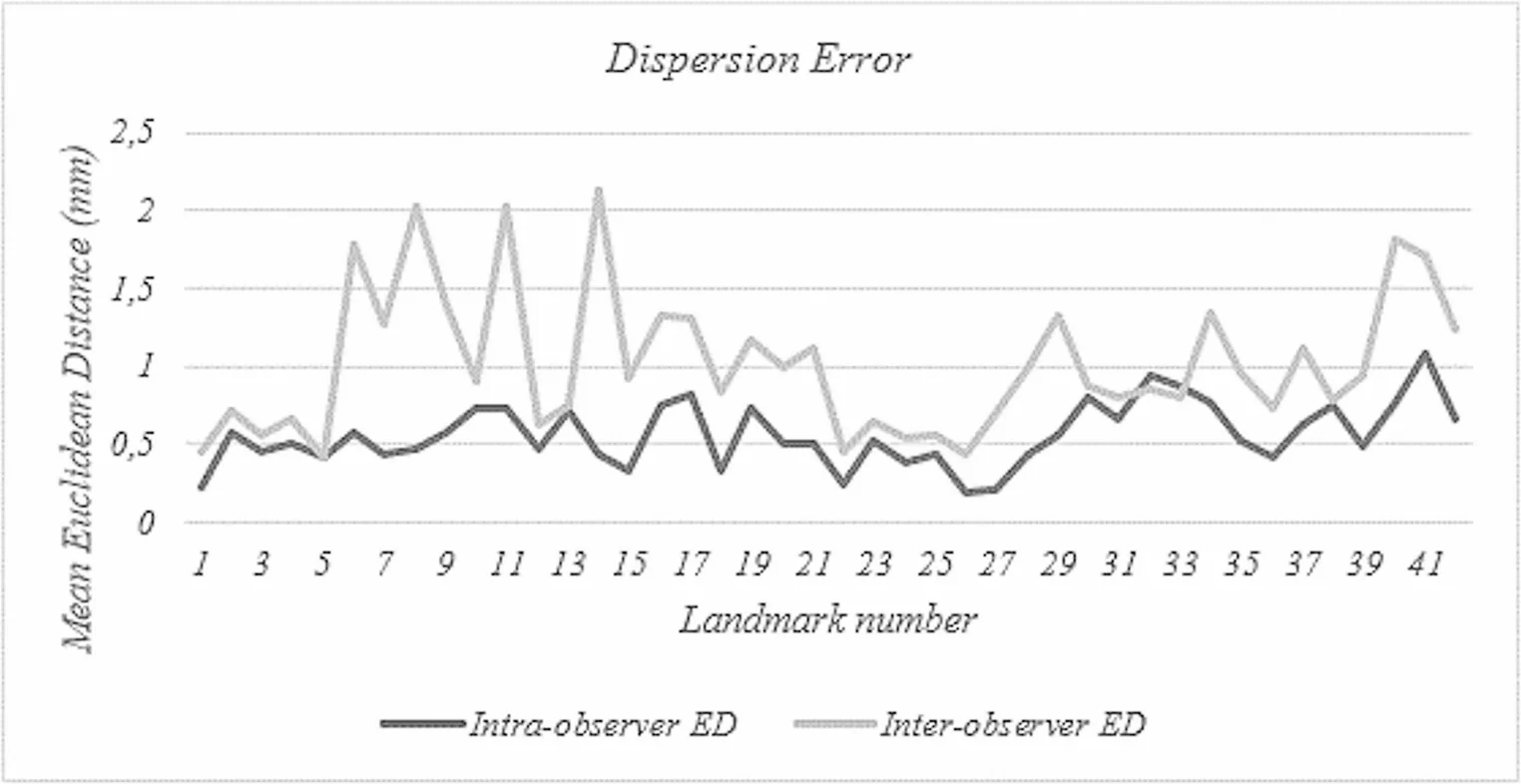
Comparison of the dispersion error (mean Euclidean distances) obtained for both the intra- and inter-observer error for the various landmarks.

GM analyses likewise confirmed high landmark repeatability, with intra-observer mean Euclidean distances below 2 mm and inter-observer error exceeding 2 mm at only three landmarks, by negligible margins (Table 13; Fig. 6).

**Table 13:** Intra- and inter-observer error represented by Euclidean distances to show the dispersion of repeated landmark placements

	Intra-observer Error		Inter-observer Error	
Landmark number and name	Euclidean distance (mm)	SD	Euclidean distance (mm)	SD
1. subspinale	0.23	0.10	0.45	0.20
2. most inferior nasal border (L)	0.57	0.30	0.71	0.38
3. most inferior nasal border (R)	0.45	0.31	0.56	0.29
4. alare (L)	0.50	0.31	0.66	0.40
5. alare (R)	0.41	0.25	0.41	0.21
6. zygoorbitale (L)	0.57	0.86	1.78	1.42
7. zygoorbitale (R)	0.43	0.32	1.27	0.85
8. lower orbital border (L)	0.47	0.40	2.02	1.04
9. upper orbital border (L)	0.58	0.49	1.40	0.84
10. dacryon (L)	0.74	0.44	0.90	0.53
11. ectoconchion (L)	0.73	0.65	2.03	4.60
12. dacryon (R)	0.47	0.36	0.63	0.51
13. ectoconchion (R)	0.71	0.50	0.75	0.53
14. lower orbital border (R)	0.43	0.30	2.13	1.28
15. upper orbital border (R)	0.34	0.27	0.93	0.58
16. zygion (L)	0.75	0.39	1.32	0.71
17. zygion (R)	0.82	0.60	1.31	0.82
18. frontomalare temporale (L)	0.33	0.19	0.84	0.34
19. frontotemporale (L)	0.74	0.37	1.17	0.73
20. frontotemporale (R)	0.50	0.48	1.00	0.84
21. frontomalare temporale (R)	0.51	0.16	1.12	0.66
22. nasion	0.25	0.10	0.46	0.41
23. glabella	0.52	0.33	0.65	0.26
24. bregma	0.39	0.25	0.54	0.47
25. lambda	0.43	0.17	0.55	0.37
26. opisthion	0.20	0.12	0.43	0.25
27. basion	0.21	0.15	0.69	0.37
28. FOB point (L)	0.44	0.31	1.00	1.04
29. FOB point (R)	0.55	0.35	1.33	0.89
30. radiculare (L)	0.81	0.46	0.88	0.65
31. radiculare (R)	0.66	0.48	0.81	0.60
32. asterion (L)	0.95	0.67	0.85	0.67
33. asterion (R)	0.88	0.60	0.80	0.51
34. mid malar projection point (L)	0.77	0.35	1.34	0.81
35. porion (L)	0.53	0.50	0.96	0.66
36. mastoidale (L)	0.41	0.26	0.74	0.59
37. mid malar projection point (R)	0.63	0.23	1.12	0.69
38. porion (R)	0.75	0.62	0.79	0.48
39. mastoidale (R)	0.48	0.25	0.94	0.84
40. eurion (L)	0.77	0.56	1.82	1.12
41. eurion (R)	1.08	0.41	1.71	0.92
42. opisthocranion	0.67	0.33	1.23	0.55
**Mean**	**0.56**	**0.37**	**1.02**	**0.74**

**Fig. 7:**
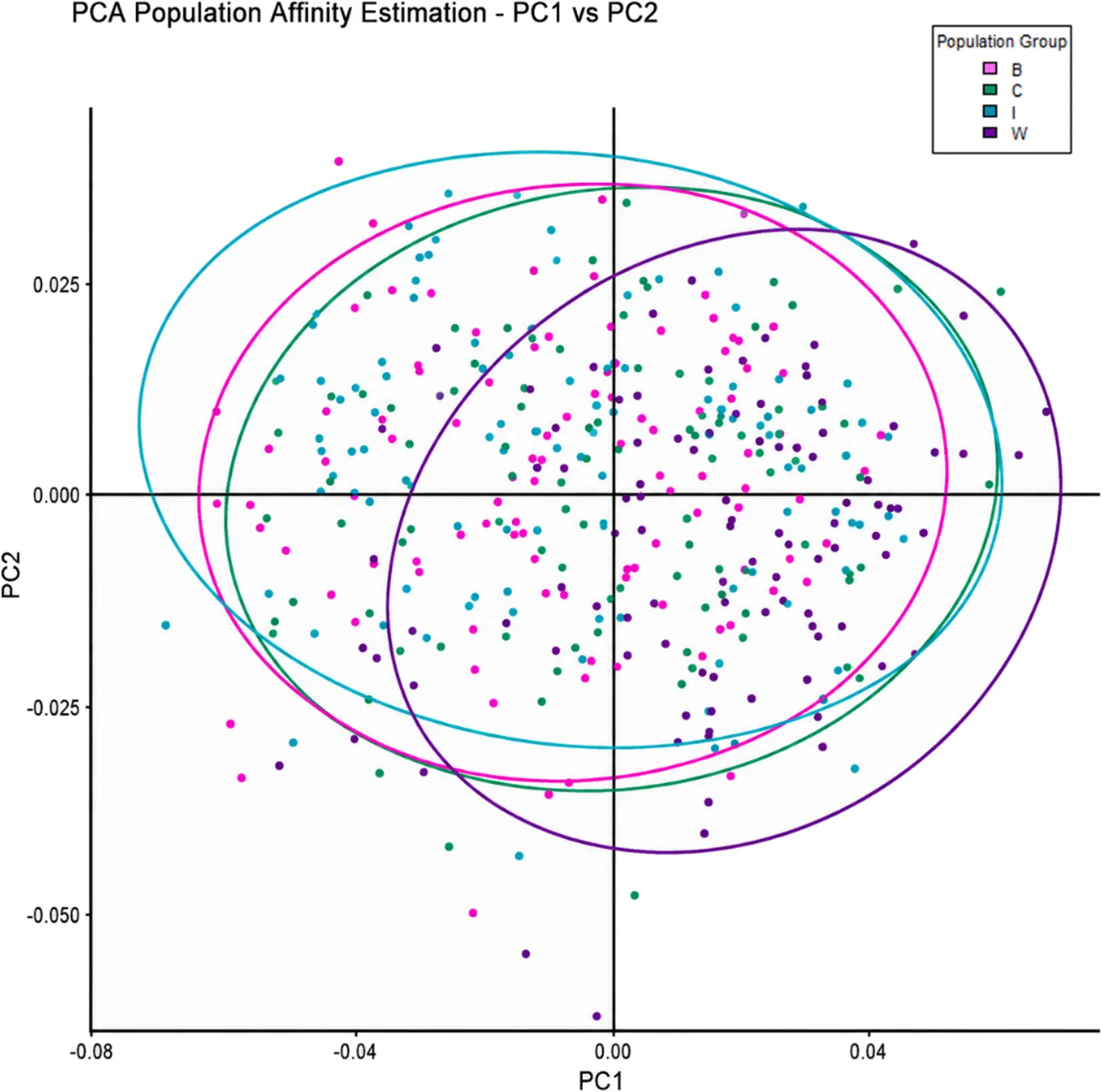
Plot of PC1 (20.1%) versus PC2 (7.3%) when assessing population affinity among Black, Coloured, Indian and White South Africans

 Following Generalised Procrustes Analysis, 22 principal components accounting for 80.56% of total shape variation were retained for further analyses (Table 14). Principal component 1 (20.1%) reflected variation concentrated in the cranial vault, while PC2 (7.3%) was associated primarily with posterior and superior cranial morphology (Fig. 7).

**Table 14:** PC score, Eigenvalues, % variance, and the cumulative variance expressed by the PC scores

PC score	Eigenvalues	% Variance	Cumulative %
PC1	0.00077990	20.112	20.112
PC2	0.00028424	7.330	27.441
PC3	0.00024504	6.319	33.760
PC4	0.00021742	5.607	39.367
PC5	0.00019371	4.995	44.362
PC6	0.00017094	4.408	48.770
PC7	0.00014137	3.646	52.416
PC8	0.00011840	3.053	55.469
PC9	0.00011262	2.904	58.373
PC10	0.00010491	2.705	61.078
PC11	0.00010140	2.615	63.693
PC12	0.00008714	2.247	65.941
PC13	0.00007775	2.005	67.946
PC14	0.00007053	1.819	69.764
PC15	0.00006434	1.659	71.424
PC16	0.00006393	1.649	73.072
PC17	0.00006196	1.598	74.670
PC18	0.00005154	1.329	75.999
PC19	0.00005014	1.293	77.292
PC20	0.00004366	1.126	78.418
PC21	0.00004260	1.099	79.517
PC22	0.00004058	1.047	80.563

 The first 22 PC scores were also assessed using ANOVA with 1000 permutations and ordinary least squares. The results showed that significant population differences were present in the data (p < 0.01) with F-value = 10.64. A further 50-50 MANOVA with distances and p-values with Holm's adjustment for multiple comparisons was run to explore the relationships among the various populations within the sample. All populations showed statistically significant differences in group mean shape (p < 0.01; Table 15), despite considerable individual-level overlap. Black South Africans showed the greatest separation in analyses, whereas Coloured and Indian South Africans were the most similar, with considerable overlap. White South Africans showed some overlap with both Coloured and Indian South Africans, although this was less pronounced than the overlap observed between Coloured and Indian South African groups.

**Table 15:** 50–50 MANOVA with distances and p-values with Holm’s adjustment for multiple comparisons

Distances between group means with *p*-values			
	B	C	I
C	0.0214 (*p* < 0.01)		
I	0.0244 (*p* < 0.01)	0.0156 (*p* < 0.05)	
W	0.0361 (*p* < 0.01)	0.0230 (*p* < 0.01)	0.0333 (*p* < 0.01)

Overall 50–50 MANOVA *p*-value < 0.01

#### Classification accuracy (GM)

**Fig. 8:**
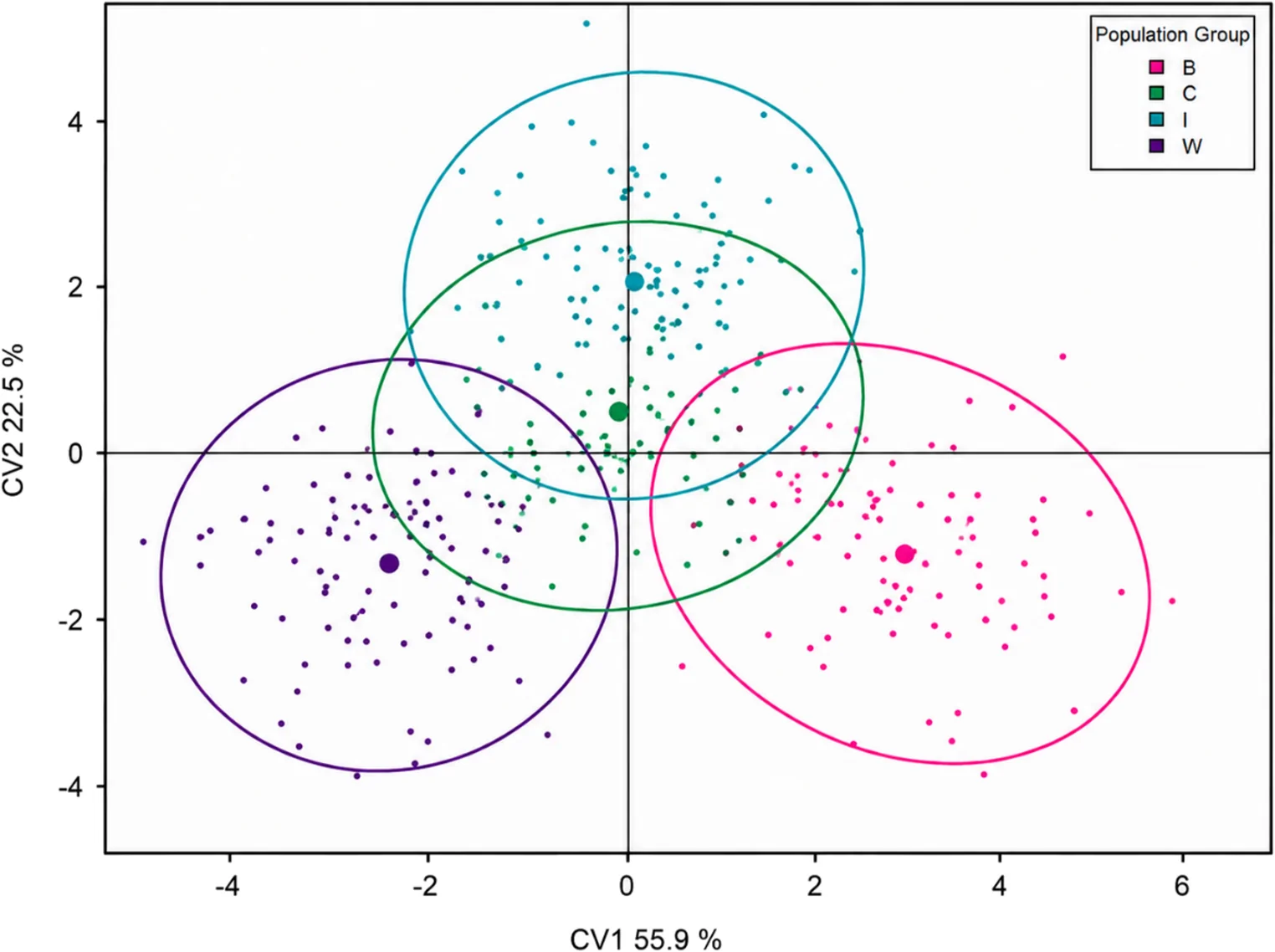
Plot of canonical variate group means and 95% confidence intervals for the CVA using ProCoords. The overall correct classification rate was 63.8%

 Canonical variate analysis (CVA) based on Procrustes coordinates produced a cross-validated classification accuracy of 63.8% for the four populations (Table 16). As with the ILD-based analysis, Black South Africans achieved the highest correct classification rates. The greatest overlap occurred among Coloured, Indian, and White South Africans, with frequent misclassification between Coloured and Indian individuals (Fig. 8).

**Table 16:** Classification matrix and correct classification rate for CVA when classifying the sample according to population using ProCoords (size-free shape variables)

From group	Into group				Correct classification
	B	C	I	W	
**B**	77	13	11	1	75.5%
**C**	12	47	23	20	46.1%
**I**	5	24	62	12	60.2%
**W**	1	17	9	75	73.5%

Total correct: 261 of 408 (63.8% cross-validated)

**Fig. 9:**
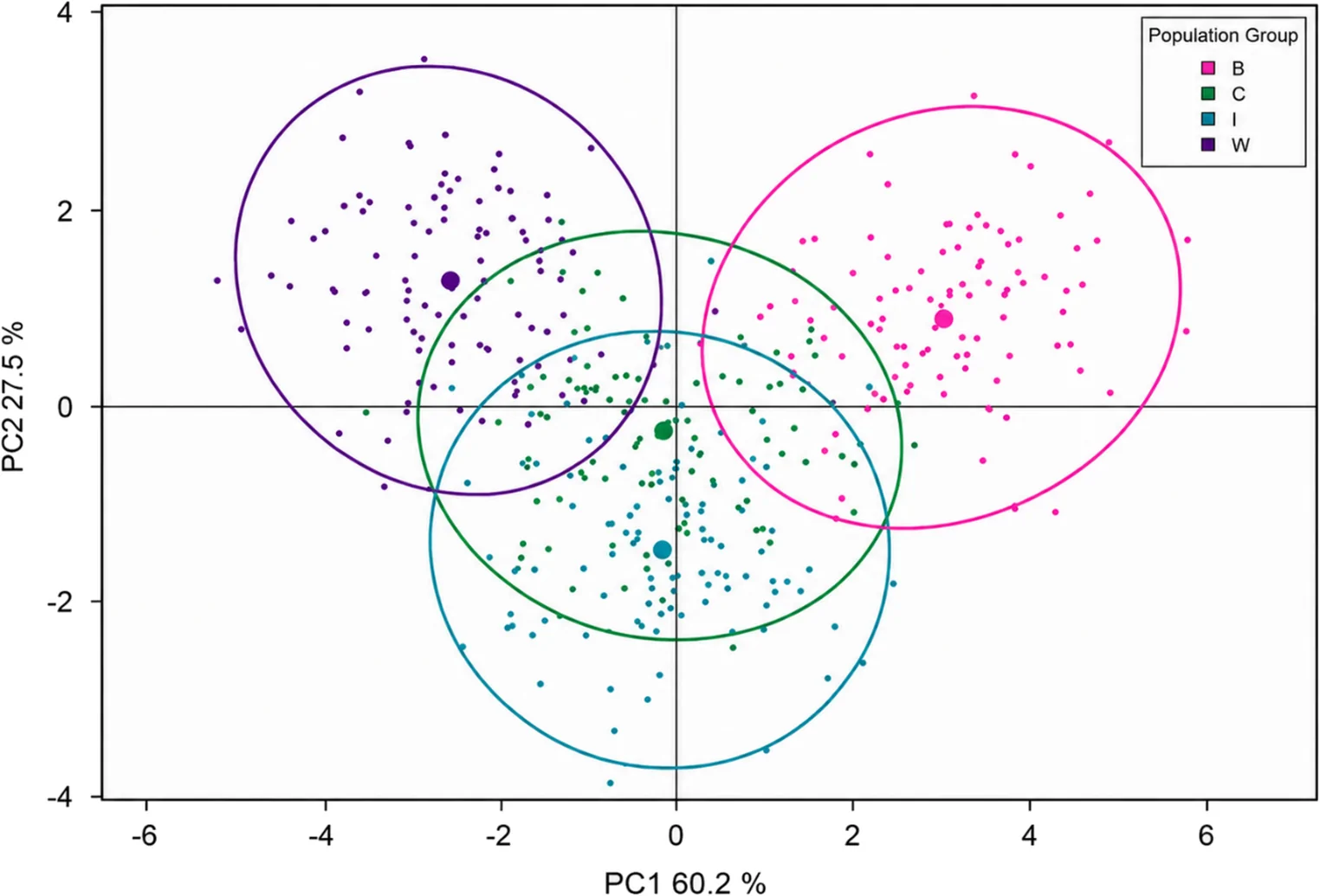
Plot of canonical variate group means and 95% confidence intervals for the CVA using ProCoord PCs. The overall classification rate was 62.6%

 Classification based on principal component scores yielded a similar overall accuracy (62.6%), mirroring the ILD results in both magnitude and pattern of misclassification (Table 17; Fig. 9).

**Table 17:** Classification matrix and correct classification rate for CVA when classifying the sample according to population using ProCoord PCs

From group	Into group				Correct classification
	B	C	I	W	
**B**	79	15	4	4	77.5%
**C**	15	39	28	20	38.2%
**I**	5	23	61	14	59.2%
**W**	1	14	10	77	75.5%

Total correct: 255 of 408 (62.6% cross-validated)

 Overall, both ILD and GM approaches identified significant cranial variation among Black, Coloured, Indian, and White South Africans. The general size-related patterns observed in the descriptive analyses were also reflected in the shape-based results, although differences in the degree of separation between groups were evident. Black South Africans consistently showed the greatest separation in analyses, while Coloured and Indian South Africans showed the greatest degree of overlap. Classification accuracies were moderate but remained above chance across analyses. These findings suggest that both cranial size and shape can detect population-level variation, although notable morphological overlap persists among certain South African populations.

## Discussion and conclusion

### Discussion

 This study explored cranial variation among contemporary South African population groups using CT-derived data. The aim was not to assess the equivalence of CT and dry bone measurements, but rather to examine whether meaningful population-level patterns could be identified within a clinically derived sample using both inter-landmark distances (ILDs) and geometric morphometric (GM) approaches. In doing so, the study contributes to a growing body of work investigating cranial variation in contemporary populations represented in medico-legal contexts.

#### Measurement repeatability

 Measurement repeatability was acceptably high for both ILDs and GM landmarks, with intra- and inter-observer error rates falling within accepted biological anthropology thresholds [17, 33, 34]. The low TEM and %TEM values, together with minimal landmark dispersion, indicate that the cranial models could be measured consistently within this study. The exclusion of a small number of less repeatable ILDs further strengthened the robustness of the analyses.

 These findings demonstrate internal measurement consistency and support the use of CT-derived cranial models for examining morphological variation within the present dataset. While previous studies have reported comparability between CT and dry bone measurements, such similarity was not tested here. Accordingly, the results should be interpreted as reflecting variation within a CT-derived sample rather than direct correspondence with skeletal collections.

#### Population-level cranial variation

 Both ILD and GM analyses revealed significant population-level differences in cranial form. Although classification accuracies were moderate (62–64%), they were well above chance and consistent with expectations given the inclusion of four biologically variable and historically admixed population groups. Patterns of misclassification were not random, but reflected structured overlap among particular groups. These structured patterns of overlap are consistent with the moderate classification rates observed and reinforce the expectation of biological continuity within historically admixed populations.

 Across analyses, Black South Africans showed the greatest separation in analyses. Inter-landmark distance results indicated relatively longer cranial lengths and broader midfacial dimensions, while GM analyses highlighted differences concentrated in nasal height–related landmarks and posterior cranial vault morphology. In contrast, White South Africans tended to display larger cranial vault and base dimensions, with a relatively rounder vault shape.

 Coloured and Indian South Africans demonstrated the greatest degree of overlap in both metric and shape-based analyses. Inter-landmark distance means placed these groups in an intermediate position between Black and White South Africans, and GM visualisations showed only subtle differences in anterior–superior facial and vault landmarks. The overlap observed between Coloured and Indian South Africans was especially pronounced in classification analyses, where misclassification frequently occurred between these two groups. White South Africans showed some overlap with both Coloured and Indian South Africans, although this was less extensive than the overlap observed between Coloured and Indian groups themselves.

 These findings align with broader anthropological evidence that craniofacial morphology reflects a combination of genetic, environmental, and developmental influences [46–49]. These findings align with broader anthropological evidence that craniofacial morphology reflects a combination of genetic, environmental, and developmental influences [46–49]. The four population groups included in this study reflect different historical migration and admixture patterns within South Africa. These population groups also reflect historical demographic and sociopolitical processes, including migration, colonial settlement, and periods of legally enforced racial segregation that influenced patterns of admixture and gene flow. Black South Africans primarily derive their ancestry from Bantu-speaking populations, whereas White South Africans are largely of European descent. Coloured South Africans represent a highly admixed population with contributions from Khoesan, Bantu-speaking African, European and Asian populations, including documented Indian ancestry, while Indian South Africans largely descend from migrants from the Indian subcontinent and have remained comparatively genetically homogeneous. These differing population histories, together with varying degrees of admixture and gene flow, likely contribute to the observed pattern of greater overlap among Coloured, Indian and White South Africans and the relative distinctness of Black South Africans [50, 51]. These findings further support the population-specific nature of cranial variation and sexual dimorphism, as demonstrated in recent CT-based studies of modern South African populations [52, 53].

 Rather than suggesting the existence of discrete biological groupings, these results highlight the importance of interpreting population affinity within a probabilistic framework. Contemporary South African populations exhibit considerable biological overlap, reflecting complex histories of migration and admixture. As such, the value of population affinity estimation lies not in definitive classification, but in its potential to contribute to a broader evidentiary framework that may assist in narrowing investigative possibilities.

 Importantly, these interpretations relate to variation observed within the present CT-derived sample and should not be taken to imply direct equivalence with skeletal-based reference data.

#### Sex-specific population affinity

 Significant sex differences were identified for the majority of ILDs and in GM shape analyses, supporting the decision to conduct sex-specific population affinity estimation. When analysed separately, females generally yielded higher classification accuracies than males. Despite these differences in performance, the same overall pattern of group separation and overlap was maintained across sexes: Black South Africans remained the most clearly differentiated group, while Coloured and Indian South Africans demonstrated the greatest degree of overlap.

 The presence of significant sex-by-population interactions for multiple ILDs further addresses the importance of applying sex-specific standards in forensic analyses. Population affinity estimation that does not account for sexual dimorphism may obscure meaningful variation or reduce classification performance.

#### Implications for forensic anthropology in South Africa

 This study highlights the value of examining contemporary cranial variation using clinically derived datasets. While traditional skeletal collections remain central to forensic anthropology, modern imaging archives offer an additional source of data reflecting present-day population structure. The findings here demonstrate that CT-derived cranial models capture patterned variation across South African groups.

 Both ILD and GM approaches produced similar classification accuracies, demonstrating that traditional craniometric methods remain valuable alongside more advanced geometric techniques. Rather than replacing established metric approaches, GM analyses complement them by modelling shape variation in a continuous framework and visualising morphological trends. The comparable performance of the two methods suggests that forensic practitioners may employ either approach, depending on available resources and case-specific requirements.

 The moderate classification rates observed here should be interpreted within context. With four population groups and documented admixture, perfect separation is neither expected nor biologically realistic. These findings should therefore be interpreted as reflecting patterns of likelihood rather than categorical assignment. Overlap between groups is expected in an admixed population, and misclassification is not indicative of methodological failure but of underlying biological continuity.

 When used cautiously and in conjunction with other elements of the biological profile, probabilistic assessments of population affinity may still contribute meaningfully toward presumptive identification in cases where other demographic information is unavailable. In this context, population affinity estimation functions not as a tool for definitive classification, but as one component of a cumulative identification process.

### Limitations

 Several limitations should be considered. Variation in CT acquisition parameters across hospitals may introduce subtle differences in image resolution, although strict inclusion criteria were applied to minimise this effect. Population affinity was inferred in some cases using surname-based methods where hospital records were incomplete. While this approach has precedent in retrospective research and was applied cautiously, it does not constitute definitive classification and may introduce misclassification bias.

Furthermore, the population categories employed reflect socially and historically defined groupings used within the South African medico-legal system. As with all population affinity estimation research, these categories do not equate to discrete biological entities. Instead, they serve as pragmatic proxies for patterned biological variation within a specific sociocultural context.

## Conclusion

 This study demonstrates that CT-derived cranial data can be used to investigate population-level cranial variation within a contemporary South African sample in a reliable and practical way. Both ILD and GM analyses identified significant differences among Black, Coloured, Indian, and White South Africans, with cross-validated classification accuracies between 62% and 64%. Black South Africans were consistently the most differentiated group, while Coloured and Indian South Africans showed the greatest morphological overlap, with White South Africans overlapping with both groups to a lesser extent. These moderate classification rates reflect the complexity of human biological variation and are consistent with expectations for historically admixed populations, reinforcing that population affinity is best understood in probabilistic rather than discrete terms.

 The inclusion of Indian South Africans helps address a longstanding gap in South African forensic reference standards and contributes toward a more representative framework for interpreting population variation. Rather than offering definitive classifications, population affinity estimation should be viewed as providing contextual biological information that may assist in presumptive identification. The findings also highlight the importance of developing modern datasets that reflect individuals more likely to be encountered in current forensic casework, as reliance solely on historical skeletal collections may limit the relevance of anthropological assessments in populations shaped by recent demographic change. By utilising clinically derived CT datasets, this study demonstrates a practical approach to expanding forensic reference frameworks beyond the limitations of historical skeletal collections and toward data that more accurately represent contemporary South African population structure.

## Data Availability

The datasets generated and/or analysed during the current study are not publicly available due to ethical and institutional restrictions but are available from the corresponding author on reasonable request.
